# Multimodal Noninvasive Assessment of C-Reactive Protein for Systemic Inflammation in Adults: Cross-Sectional Study

**DOI:** 10.2196/77108

**Published:** 2025-08-26

**Authors:** Jinjoo Shim, Sinziana Muraru, Vanessa Löw, Caroline Evers, Sandro Riedo, Rucsandra Dobrota, Elgar Fleisch, Oliver Distler, Filipe Barata

**Affiliations:** 1 Centre for Digital Health Interventions, ETH Zurich, Zurich, Switzerland; 2 Department of Biostatistics, Harvard T.H. Chan School of Public Health, Harvard University, 677 Huntington Avenue, Boston, MA, 02115, United States, 1 6174321056; 3 Department of Rheumatology, University Hospital Zurich, University of Zurich, Zurich, Switzerland; 4 Centre for Digital Health Interventions, University of St Gallen, St Gallen, Switzerland

**Keywords:** digital biomarker, noninvasive biomarker, C-reactive protein, CRP, systemic inflammation, remote monitoring, chronic inflammatory disease, biosensors, urine, sweat, saliva, exhaled breath, core body temperature, stool, interleukin, IL-1β, IL-6, IL-8, IL-10, tumor necrosis factor, TNF-α

## Abstract

**Background:**

Accurate and accessible measurements of inflammatory biomarkers are crucial for the diagnosis and monitoring of inflammatory diseases. The gold-standard C-reactive protein (CRP) requires venipuncture, which, despite providing high-quality samples, can cause discomfort, anxiety, and pain, particularly in vulnerable populations such as older patients. It is also resource-intensive, is unsuitable for remote or at-home use, and lacks continuous monitoring capability. These limitations limit patient autonomy and self-management, potentially leading to poorer prognosis due to delays in assessment and medical treatments. As digital health technologies advance, there is increasing interest in leveraging digital biomarkers for remote and real-time monitoring of systemic inflammation. Digital biomarkers derived from noninvasive biofluids could provide a scalable solution for tracking inflammatory status, offering a patient-centered alternative to traditional blood-based assessments. To date, however, there is no consensus on the most suitable modality for assessment or its digitization potential. Therefore, a comprehensive evaluation of the feasibility, reliability, and patient acceptability toward noninvasive, digital inflammatory biomarkers is needed.

**Objective:**

Our aim is to evaluate the feasibility of various noninvasive methods to assess inflammatory markers and identify the optimal modality for predicting serum CRP levels.

**Methods:**

Inflammatory biomarkers were assessed in 20 participants (10 patients with systemic inflammation defined as a CRP level >5 mg/L and 10 controls) using 6 noninvasive samples (urine, sweat, saliva, exhaled breath, core body temperature, and stool samples) alongside serum samples. Patient preferences were retrieved via a questionnaire. Mann-Whitney *U* test, Spearman correlation, and all-subset regression were conducted to assess the relationships between serum and nonserum biomarkers and identify optimal predictive models for serum CRP levels.

**Results:**

CRP levels were significantly elevated in the inflammation group compared to controls in urine (median 4.5, IQR 4.15‐10.3 vs median 0.69, IQR 0.24‐1.39 μg/mmol; *P*=.001) and saliva (median 4910, IQR 2735-13,275 vs median 473, IQR 309-700 pg/mL; *P*=.001). Urine and saliva CRP levels strongly correlated with serum CRP (*r*_sp_=0.886; *P*<.001; *r*_sp_=0.709; *P*<.001). The multimodal model using urine and saliva CRP predicted serum CRP levels with 76.1% outperforming single-modality models. Patients favored urine and saliva tests over blood tests.

**Conclusions:**

Urine and saliva represent promising noninvasive alternatives to traditional blood tests for assessing CRP, enabling more accessible and less invasive diagnostic and monitoring approaches.

## Introduction

The diagnosis, assessment, and ongoing monitoring of systemic inflammatory diseases rely on accurate and readily available measurements of inflammatory biomarkers. Among these, the C-reactive protein (CRP) is extensively used to assess patients’ inflammatory state and inform clinical decision-making due to its stability, long half-life, and lack of circadian variability [1]. In rheumatoid arthritis (RA), for example, disease activity and treatment response are assessed based on the levels of CRP (or erythrocyte sedimentation rate) in combination with physical assessment such as inflamed or tender joint counts [2,3].

In current practice, CRP levels are measured in blood samples. While providing high-quality results, venipunctures are associated with distress and anxiety and cause pain and discomfort for patients [4,5]. This issue is particularly pronounced in vulnerable populations, such as children or older people, who report significantly higher levels of distress during venipuncture procedures [6,7]. In individuals with difficult venous access, venipuncture may require multiple attempts, increasing the procedure risks [8].

Blood tests also incur high costs due to the need for trained personnel and specialized laboratory equipment for sample collection and analysis. Furthermore, repeated measurements are impractical in remote settings, limiting patient autonomy and posing substantial challenges in patient-centered care. The lack of timely, accessible monitoring may delay assessments and medical treatments, potentially leading to a poorer prognosis.

At present, consumer-accessible CRP measurement devices remain scarce. Point-of-care devices for the CRP test, priced between US $500 and US $2000 per unit, are designed for health care professionals and still require invasive blood samples. These devices are not only costly and bulky but also have limited sensitivity for CRP assessment, making them impractical for individual use in remote settings. As the global burden of systemic inflammatory diseases continues to rise [9], the development of noninvasive CRP monitoring is essential for improving disease monitoring and patient care.

As digital health technologies advance, there is increasing interest in leveraging digital biomarkers for remote and real-time monitoring of chronic conditions. To date, however, no noninvasive and digital biomarker for the assessment of CRP has been developed. Recent studies have demonstrated that noninvasive measurement of CRP and cytokines in nonblood biofluids such as saliva, urine, and sweat is generally feasible. While CRP has been quantified in urine, most research is focused on urologic conditions [10-13], and its relevance in systemic inflammatory diseases remains largely unexplored. Salivary CRP levels have been correlated with serum CRP in healthy adults and across cardiovascular conditions, chronic obstructive pulmonary disease, or depression [14-17]; however, data on salivary CRP in chronic inflammatory conditions are still limited [18,19]. Wireless wearable biosensors have also shown potential in detecting CRP and inflammatory cytokines in sweat [20,21].

For chronic inflammatory diseases with organ-specific manifestations such as asthma and inflammatory bowel disease, well-established biomarkers such as fractional exhaled nitric oxide (FeNO) and fecal calprotectin are available [22,23], but their relevance in assessing inflammation in other inflammatory diseases has yet to be thoroughly investigated. Moreover, recent studies have used continuous monitoring of core body temperature (CBT) via noninvasive digital sensors to explore circadian rhythmicity and thermoregulation, which might be disrupted in patients with systemic inflammation (SI) [24].

Despite this progress in developing noninvasive methods for the measurement of inflammatory markers, there is no consensus on the most suitable modality for their assessment and for the digitization potential. Existing studies have primarily focused on 1 or 2 biofluids without conducting multimodal measurements and a comprehensive comparison to identify the best-suited noninvasive modality for assessing inflammation. Furthermore, no study has examined patients’ preferences for different measurement methods. Evidence suggests that preferences and adherence significantly impact health outcomes, treatment effectiveness, and patient well-being, which may critically impact the real-world applications [25].

Given the challenges associated with blood-based CRP measurement, the growing interest in noninvasive alternatives, and the potential of emerging digital solutions for remote monitoring, there is a need for a comprehensive evaluation of their feasibility, reliability, and patient acceptability.

To address this limitation, our study aims to investigate (1) whether CRP can be detected in noninvasive methods and (2) whether noninvasive CRP correlates significantly with serum CRP levels. To this end, we set out this proof-of-concept study to simultaneously collect and measure inflammatory biomarkers from 6 different noninvasive methods and evaluate their associations with serum biomarkers.

By identifying the optimal noninvasive surrogate markers for serum CRP, our study could guide further efforts in the development of scalable digital health monitoring solutions or digital inflammatory biomarkers, supporting an efficient and patient-centered personalized care.

## Methods

### Study Population and Recruitment

Our target population included patients with elevated inflammatory parameters (serum CRP >5 mg/L) due to flares across a variety of systemic inflammatory diseases. This approach enhances the generalizability of our findings, ensuring that the noninvasive biomarkers are applicable across different diseases. Patients admitted to the rheumatology ward of the University Hospital Zurich (USZ), Switzerland, for planned or emergency investigations were prescreened by a study physician for having CRP >5 mg/L. If deemed eligible and not needing immediate anti-inflammatory treatment, the patients were informed about our study. The control group was recruited the same way from the rheumatology ward from patients not having increased CRP levels (CRP≤5 mg/L). All participants were given 24 hours or overnight to consider study participation before providing informed consent. Both study visits occurred during the hospital stay, avoiding additional travel burden for the patients. The recruitment procedure is presented in Figure S1 in Multimedia Appendix 1 and further detailed in the study protocol [26].

We included 10 adult patients with SI and 10 control participants. Patients fulfilled the classification criteria of the respective inflammatory disease. We excluded patients requiring initiation of a new treatment with glucocorticoids or immunosuppressive medication, patients diagnosed with active psychiatric disorders, or patients having language barriers or other factors impairing adherence to the study protocol. Moreover, samples with signs of urinary tract infection were excluded from the analysis to reduce the risk of false positive results related to local rather than SI and ensure the reliability of urinary CRP as a potential biomarker in this limited sample size. Detailed exclusion criteria are presented in Figure S1 in Multimedia Appendix 1.

### Sample Procedures, Sample Collection, and Measurement

Figure S2 in Multimedia Appendix 1 describes the study procedure. Participants were asked to fast for at least 2 hours prior to the study visit and avoid drinking other liquids than water, using chewing gums, brushing teeth, mouthwash, or smoking.

At the first visit, we applied a questionnaire assessing demographic information and history regarding oral health as well as recent surgical procedures and hospitalizations (Methods S1 in Multimedia Appendix 1). Venous blood samples for biomarker analysis were collected (1 heparin gel tube for CRP and 1 native tube for the immunological parameters), followed by the collection of midstream urine samples. A dipstick test (ie, Roche Combur Test Strip) to screen for signs of urinary tract infection was performed immediately after urine collection. Saliva samples were obtained using an absorbent swab (Salimetrics SalivaBio Oral Swab), placed under the tongue for 3 minutes, and disposed directly into a collection tube. Two sweat patches (ie, PharmChek Sweat Patch) [21] were applied to participants’ arms or abdomen for sweat collection.

A CBT sensor (greenTEG CaleraResearch CORE) was then placed on the upper body, approximately 20 cm below the armpit using a medical patch. Calera Research CORE is a heat flux-based thermometer calculating body temperature based on a clinically validated algorithm using built-in heat flux and skin temperature sensors [27]. We focused on nighttime hours to mitigate the potential impact of external factors such as participation in rehabilitation programs or physical activity on patients’ core temperature rhythm during daytime.

FeNO measurements were conducted using the Bosch Vivatmo me device [28]. Finally, we provided instructions for stool sampling. To minimize potential confounding from the diurnal variation of CRP levels, all first visits were conducted within a restricted time window between 11 AM and 3 PM.

At the second visit after 48 hours, the researcher collected the CBT sensor, sweat patches, and stool sample. Participants were asked to rank their preference for sampling methods, including a blood test and 6 noninvasive tests, using an ordinal ranking method. They ranked each test on a scale from 1=most preferred to 7=least preferred. Questionnaires were applied in German or English as preferred by patients (Methods S2 and S3 in Multimedia Appendix 1 for English version; the German version is available upon request).

A part of the collected urine (10‐20 mL) was retained for analysis in the external laboratory and stored at −80 °C until analysis. The remaining urine as well as the blood samples were centrifuged at room temperature at 3710 rpm for 10 minutes (5 minutes for urine), aliquoted into cryovials, and stored at −80 °C until analysis. Stool, saliva, and sweat patches were frozen within 1 hour from collection and stored at −80 °C until analysis. In the external laboratory, the urine samples were thawed and centrifuged for 15 minutes at 1000× *g* at 2‐8 °C for preparation. Clinical data were gathered from electronic medical records, including anthropometric measurements (eg, weight, height, and BMI), laboratory results (eg, erythrocyte sedimentation rate), disease characteristics (eg, diagnosis of inflammatory disease, disease duration, and comorbidities), medication (eg, type, duration, and dose), and relevant measures of disease activity. Urine CRP was analyzed both as an absolute concentration and as a creatinine-normalized ratio to account for urine dilution. Specifically, we standardized urine CRP measurements according to urine creatinine levels, following established protocol [29]. Comorbidities were retrieved from the electronic records.

### Immunoassays

Analysis of serum CRP, serum and urine total protein, albumin, creatinine, and stool calprotectin was conducted at the Institute of Clinical Chemistry of the USZ. Saliva, urine, and sweat CRP and cytokine analyses were performed at Swiss BioQuant AG. Serum cytokines were analyzed at the Department of Immunology of the USZ. Biomarkers with a detection rate below 70% were excluded from the analysis to ensure the reliability of the findings.

Serum CRP levels were assessed using an immunoturbidimetry assay conducted on a Cobas 8000 c702 system (Roche Diagnostics). Saliva, urine, and sweat CRP were determined via enzyme-linked immunosorbent assay (Salimetrics, Abcam, and Creative Diagnostics), following manufacturer protocols [30-32]. Readings for saliva, urine, and sweat CPR analyses were obtained with a SpectraMax i3 multimode microplate spectrophotometer (Molecular Devices), with a wavelength set to 450 nm.

Serum and urine concentrations of the total amount of protein, albumin, and creatinine were quantified with the immunoturbidimetry method (Cobas 8000). Stool calprotectin levels were determined using chemiluminescence immunoassay (Liaison XL, DiaSorin).

Serum levels of interleukin (IL)-1β, IL-6, and tumor necrosis factor (TNF)-α were quantified using a Luminex Magpix analyzer (Luminex Corporation). IL-8 in serum was analyzed using an enzyme-linked immunosorbent assay (R&D Systems). IL-10 in serum was assessed using a cytometric bead array (BD Biosciences). IL-6, IL-8, IL-10, IL-1β, and TNF-α in sweat and urine samples were analyzed using MSD V-PLEX plates and read with QuickPlex SQ 120 electrochemiluminescence reader (MSD, Meso Scale Discovery).

### Statistical Analysis

In the descriptive analysis, we presented continuous variables with median and IQR and categorical variables with count and percentage. We used the Mann-Whitney *U* test and the chi-square test (or Fisher exact test for expected counts<5) for univariable analysis. We tested the normality of distribution using the Shapiro-Wilk test. Comparisons of end points between SI and control groups were visualized using boxplots and assessed with the Mann-Whitney *U* test. Spearman correlation coefficient (*r*_sp_) evaluated correlations between serum and nonserum biomarkers as well as between biomarkers and clinical variables.

We further assessed the predictive accuracy of multimodal, noninvasive biomarkers using all-subset regressions to identify the best model for predicting the serum CRP level among all possible combinations. This method systematically evaluates all possible predictor combinations for each full model and selects the fitted model with the optimal criterion value among candidate models based on the adjusted *R*^2^ value. *R*^2^ represents how much of the variation in the dependent variable of the model is explained by variation in its predictors [33]. An *R*^2^ of 0 indicates the prediction is impossible based on input variables, whereas an *R*^2^ of 1 represents a perfect prediction with no unexplained variability. Generally, an *R*^2^ above 0.6 indicates an algorithm to be useful, and an *R*^2^ above 0.7 would be considered a substantial predictive ability. All statistical tests were 2-sided, and a significance level of .05 was considered. Statistical analyses were performed using R (version 4.3.1; R Foundation for Statistical Computing). The study design and reporting followed the STROBE (Strengthening the Reporting of Observational Studies in Epidemiology) statement [34].

### Sample Size Calculation

Because there is no prior evidence of the outcome measures in this context, we calculated the sample size based on studies of adult patients diagnosed with RA, which is one of the most prevalent systemic inflammatory diseases. To ensure the detection of even small differences in CRP within the lower range of the inflammatory spectrum, we adopted a conservative approach and based our sample size calculation on a population of patients with RA having low disease activity. For instance, Bechman et al [35] indicated serum CRP levels in patients with RA during flares ranging from 5 to 9 (SD 3.0) mg/L. Considering these results, it is plausible to expect a difference of 3 to 6 mg/L in serum CRP levels between SI versus non-SI states among patients with RA. On this basis, assuming 80% of power, 5% of type I error, and equal variance in groups, we determined that a sample size of 10 participants per group is required to detect a 4 mg/L difference in serum CRP levels with an SD of 3.0. Therefore, we aimed to complete data collection for a total of 20 participants.

### Ethical Considerations

The study was conducted in accordance with the Declaration of Helsinki, the principles of Good Clinical Practice, the Human Research Act, and the Human Research Ordinance [36,37] and after receiving approval from the independent ethics committee of the Canton of Zurich (BASEC-Nr: 2022‐01386) on October 28, 2022. Written patient information and consent documents were available in both English and German. All participants provided written informed consent before being included in the study. In addition to the primary data analysis, the consent included a clause allowing for the encrypted data and biological material collected during this project to be stored and reused for future research. They were also informed of their right to withdraw consent at any time without providing justification. All project data were stored and handled with strict confidentiality. At inclusion, participants were assigned a study-specific unique identifier to be used on case report forms and further data analysis. Clinical data were stored on a password-protected server at the USZ, accessible only through the unique identifier provided by the USZ research team. The participant identification list is securely stored at USZ and will be destroyed 10 years after project completion. FeNO and questionnaire data containing the unique identifier were saved on a password-protected network drive at ETH Zurich, with access limited to authorized personnel and monitored through access logs. No images or visual materials included in this paper contain identifiable participant information. Participants were compensated with a voucher for 30 CHF (US $37) for their involvement in the study.

## Results

### Characteristics of Patients With SI and Controls

In total, 20 participants were enrolled in the study, 1 of whom was excluded due to a urinary tract infection, resulting in 19 participants included in the analysis. Of these 19, a total of 4 had urine CRP concentrations below the assay’s lower limit of quantification.

The baseline characteristics of 19 participants are presented in Table 1. Demographic and baseline characteristics were similar between SI and controls. The levels of creatinine, albumin, and total protein in serum and urine were also comparable.

**Table 1.** Baseline demographic, clinical, and laboratory characteristics of the 19 study participants.

Variables	SI^a^ (n=10)	Control (n=9)	*P* value
Age at recruitment (years), median (IQR)	56 (38.0-63.0)	57 (47.0-59.0)	.68
Gender, n (%)			>.99
Women	7 (70)	6 (67)	
Men	3 (30)	3 (33)	
Employment status, n (%)			.72
Full-time	5 (50)	3 (33)	
Part-time	2 (20)	3 (33)	
Else	3 (30)	3 (33)	
Education, n (%)			.37
College or above	4 (40)	1 (11)	
Else	6 (60)	8 (89)	
BMI category, n (%)			.93
Underweight or normal (<25 kg/m^2^)	2 (20)	2 (22)	
Overweight (25‐29.9 kg/m^2^)	3 (30)	2 (22)	
Obese (≥30 kg/m^2^)	5 (50)	5 (56)	
Smoking, n (%)			>.99
Previous or current smoker	2 (20)	2 (22)	
No	8 (80)	7 (78)	
Comorbidities, n (%)			
Arterial hypertension	4 (40)	4 (44)	>.99
Chronic pain syndrome	4 (40)	8 (89)	.08
Depression	2 (20)	2 (22)	>.99
Prior history, n (%)			
Had hospitalization within 1 year	2 (20)	0 (0)	.50
Had operation within 1 year	2 (20)	0 (0)	.50
Oral health, n (%)			
Fasted more than 2 hours	4 (40)	7 (78)	.23
No brushing teeth within 2 hours	10 (100)	9 (100)	N/A^b^
No chewing gum within 2 hours	10 (100)	9 (100)	N/A
No smoking within 2 hours	8 (80)	9 (100)	.50
No drinking within 24 hours	9 (90)	9 (100)	>.99
No bleeding gums within 24 hours	10 (100)	9 (100)	N/A
No current oral diseases	7 (70)	8 (89)	.66
Laboratory parameters, median (IQR)			
Serum creatinine (mmol/L)	81.0 (74.3-96.3)	75.0 (67.0-89.0)	.46
Serum albumin (g/L)	39.5 (38.0-40.0)	44.0 (41.0-44.0)	.003
Serum total protein (g/L)	70.5 (67.3-74.0)	72.0 (69.0-74.0)	.62
Urine creatinine (mmol/L)	4.3 (3.4-5.5)	5.9 (4.5-11.4)	.12
Urine albumin/creatinine ratio (mg/mmol)	0.84 (0.58-1.53)	1.02 (0.67-2.45)	.41
Urine total protein/creatinine ratio (g/mmol)	10.74 (8.59-14.73)	8.91 (7.17-14.08)	.81

a SI: systemic inflammation.

b N/A: not applicable.

Patients with SI had various diagnoses, ranging from arthritis to adult-onset Still disease and giant cell arteritis (Table 2).

The median age at the onset of the inflammatory disease and disease duration were 55.5 (IQR 32.3-60.5) and 1.25 (IQR 0.5-5.3) years, respectively.

Control group patients were inpatients who attended a program for chronic musculoskeletal pain, including chronic back pain and fibromyalgia. Among them, 6 had no inflammatory disease. The most common comorbidity, like the SI group, was obesity. In total, 3 patients had an inflammatory disease in remission under treatment. Detailed diagnoses and treatment information for these 3 patients are provided in Table S5 in Multimedia Appendix 1.

**Table 2.** Inflammatory disease diagnosis, duration, and main anti-inflammatory medications at the time of inclusion for participants with systemic inflammation.

Inflammatory disease	Disease duration (years or diagnosed at inclusion)	Main medication for inflammatory diagnosis at inclusion
Mixed crystal-induced arthropathy (CPPD^a^ and gout)	6	Prednisone
Transient polyarthritis	Diagnosed at inclusion	No DMARDs^b^
Seropositive erosive rheumatoid arthritis	20	Prednisone
Inflammatory syndrome likely linked to multiple sclerosis	1.5	Ocrelizumab
VEXAS^c^ syndrome	0.5	Ruxolitinib, prednisone
Pigmented villonodular synovitis and metabolic syndrome	0.5	No DMARDs
Seronegative polyarthritis	3	NSAID^d^; no DMARDs
Adult-onset Still disease	0.3	NSAID; no DMARDs
Peripheral spondyloarthritis	9	Certolizumab pegol
Giant cell arteritis	1	Abatacept, methotrexate, prednisone

a CPPD: calcium pyrophosphate crystal deposition disease.

b DMARD: disease-modifying antirheumatic drug.

c VEXAS: vacuoles, E1 enzyme, X-linked, autoinflammatory, somatic.

d NSAID: nonsteroidal anti-inflammatory drug.

### Analysis of the Primary End Point

The median serum CRP level in patients with SI and controls was 18 (IQR 9.3-68.8) and 1.3 (IQR 1.2-1.8) mg/L, respectively (*P*<.001). CRP detection rates were 100% (19/19) for serum and saliva, and 79% (15/19) for urine. Four urine samples (1 among the SI group and 3 controls) had CRP levels below the lower limit of quantification for urine (31.3 pg/mL), whereas CRP levels for these individuals were detectable in both serum and saliva (Table S4 in Multimedia Appendix 1). The lower detection rate in urine may be attributed to its higher limit of quantification (31.3 pg/mL), compared to 0.6 mg/L for serum and 25.0 pg/mL for saliva, indicating greater sensitivity in the latter 2 biofluids. CRP could not be detected in sweat samples.

**Figure 1.**
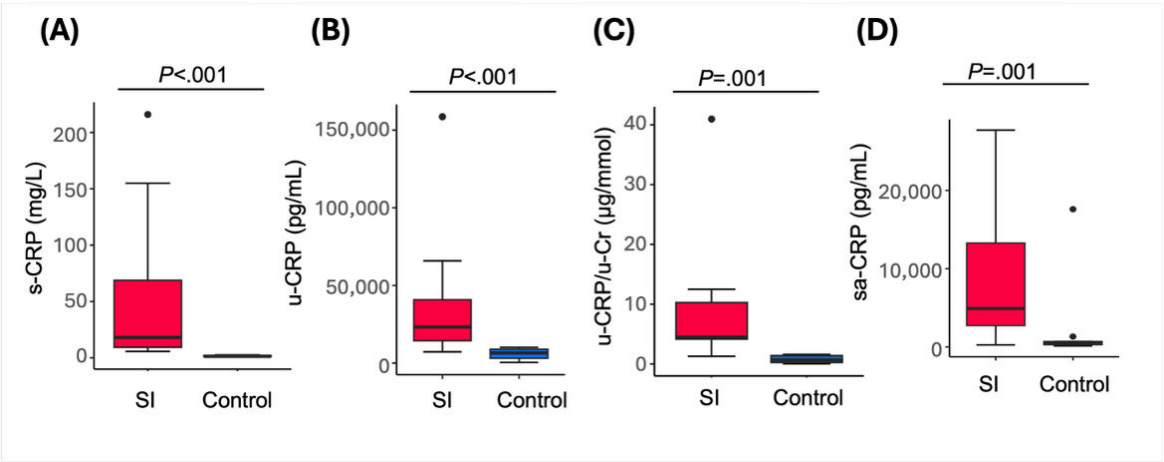
Distributions of CRP levels in serum and noninvasive biofluids among participants with SI and controls. (A) s-CRP, (B) u-CRP, (C) u-CRP/u-Cr, and (D) sa-CRP. Boxes indicate the IQR, and the horizontal line within each box represents the median, and whiskers denote 1.5×IQR. Dots represent outliers. The *P* values are based on the Wilcoxon test. CRP: C-reactive protein; s-CRP: serum C-reactive protein; sa-CRP: saliva C-reactive protein; SI: systemic inflammation; u-CRP: urine C-reactive protein; u-CRP/u-Cr: creatinine-normalized urine C-reactive protein.

CRP levels were significantly higher in the serum, urine, and saliva of patients with SI compared to controls (Figure 1). The median creatinine-adjusted urine CRP was significantly higher in patients with SI compared to controls: 4.5 (IQR 4.15-10.3) versus 0.69 (IQR 0.24-1.39) μg/mmol, respectively (*P*=.001). The absolute values are presented in Table S1 in Multimedia Appendix 1.

Similarly, median saliva CRP levels were substantially elevated in patients with SI compared to controls: 4910 (IQR 2735-13,275) versus 473 (IQR 309-700) pg/mL, respectively (*P*=.001).

**Figure 2.**
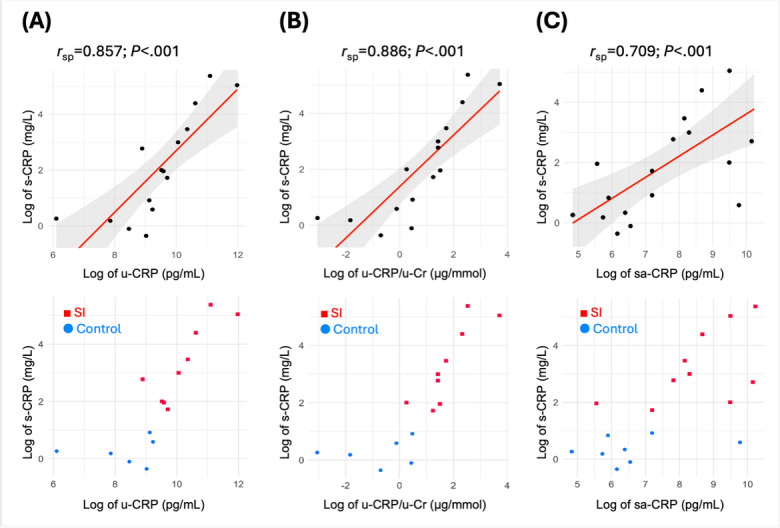
Correlations between s-CRP and non–s-CRP levels. (A) s-CRP and u-CRP, (B) s-CRP and u-CRP/u-Cr, and (C) s-CRP and sa-CRP. Each top panel displays a scatterplot of log-transformed values with a linear regression line (red) and 95% confidence band (gray). Bottom panels show individual data points stratified by group: participants with SI (red squares) and controls (blue circles). CRP: C-reactive protein; *r*_sp_: Spearman correlation coefficient; s-CRP: serum C-reactive protein; sa-CRP: saliva C-reactive protein; SI: systemic inflammation; u-CRP: urine C-reactive protein; u-CRP/u-Cr: creatinine-normalized urine C-reactivproteinn..

We assessed the correlations between serum and nonserum CRP levels (Figure 2). Creatinine-normalized urine CRP showed the strongest correlation with serum CRP (*r*_sp_=0.886; *P*<.001), followed by salivary CRP (*r*_sp_=0.709; *P*<.001). These nonserum CRP values effectively discriminated between patients with SI and controls. Therefore, noninvasive CRP measurements accurately identified active inflammation irrespective of the underlying diagnosis.

### Analysis of the Secondary and Tertiary End Points

All cytokines were detected in serum samples. IL-8 was detected in 74% (14/19) of sweat samples and 100% (19/19) of urine samples. IL-6 was detected in 42% (8/19) of sweat samples and 74% (14/19) of the urine samples. IL-10, IL-1β, and TNF-α were detected in less than 50% of the sweat and urine samples.

The median serum IL-6 level in patients with SI and controls was 2.58 (IQR 1-10.1) and 1 (IQR 1-1) pg/mL, respectively (*P*=.02). The median serum TNF-α level in patients with SI and controls was 8.85 (IQR 6.72-9.44) and 5.83 (IQR 3.4-7.17) pg/mL, respectively (*P*=.02). Serum IL-6 and TNF-α demonstrate a moderate correlation with serum CRP (*r*_sp_=0.68; *P*=.001 and *r*_sp_=0.64; *P*=.003, respectively).

**Figure 3.**
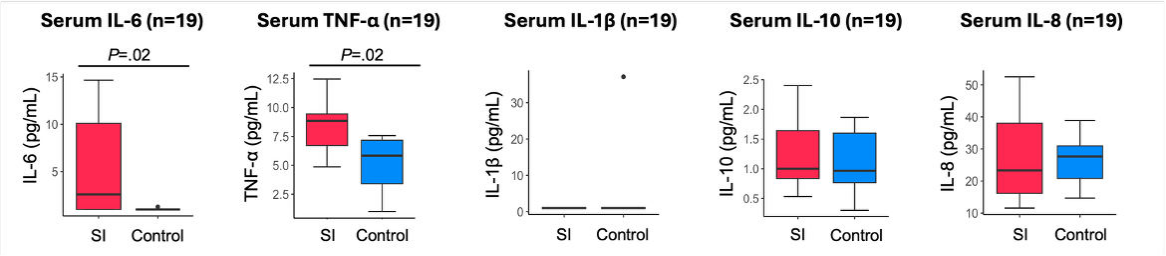
Distributions of inflammatory cytokine levels in serum among participants with SI and controls. Biomarkers with a detectability rate above 70% are shown. Boxes indicate the IQR, and the horizontal line within each box represents the median, and whiskers denote 1.5×IQR. Dots represent outliers. The *P* values are based on the Wilcoxon test. IL-1β: interleukin-1 beta; IL-6: interleukin-6; IL-8: interleukin-8; SI: systemic inflammation; TNF-α: tumor necrosis factor-alpha.

In the analysis of inflammatory cytokines, we observed significant differences in serum levels of IL-6 and TNF-α between groups (Figure 3). IL-1β, IL-10, and IL-8 levels were detectable in serum samples but did not show a statistically significant difference between groups.

In noninvasive methods, sweat IL-8, urine IL-8, and urine IL-6 showed a sufficient detectability rate; however, they did not exhibit significant differences between groups (Figure S3 in Multimedia Appendix 1).

We observed a significant difference in the FeNO levels comparing SI and controls (21 vs 11 ppb; Table S2 in Multimedia Appendix 1). However, due to the low detection rate of FeNO (8/19, 42%), the sample size was small. Moreover, we found that 3 SI patients with measurable FeNO levels had a history of respiratory inflammation, including allergic asthma and viral airway infection. Thus, we assumed that the increased FeNO levels were more likely associated with the local inflammation rather than systemic disease.

No significant difference was observed in fecal calprotectin levels between groups. We analyzed continuous, longitudinal CBT data from 13 participants (6 SI patients and 7 controls; Figure S4 in Multimedia Appendix 1). The minimum (nadir), maximum (peak), mean, and SD of the core temperature values during night hours between 10 PM and 7 AM the next day are summarized in Table 3. Overall, we did not find significant differences in CBT characteristics between patients with SI and controls. When inspecting individual cases, a notable temperature pattern emerged in the patient with adult-onset Still disease, characterized by a significant increase in nocturnal CBT (Figure S4A in Multimedia Appendix 1). This is in line with the literature describing the highest temperature spikes in the evening in these patients [38].

**Table 3.** Core body temperature (CBT) metrics in participants with systemic inflammation (SI) and controls^a^.

Diagnosis		Nadir CBT (°C)	Peak CBT (°C)	Mean CBT (SD) (°C)
Patients with SI				
1	Adult-onset Still disease	36.4	38.5	37.3 (0.708)
2	VEXAS^b^ syndrome	36.4	37.0	36.6 (0.151)
3	Peripheral SpA^c^	36.7	37.2	36.9 (0.102)
4	Undifferentiated seronegative polyarthritis	36.3	36.8	36.6 (0.117)
5	Giant cell arteritis	36.3	36.8	36.5 (0.114)
6	Multiple sclerosis	36.8	37.1	36.9 (0.062)
	SI group average	36.5	37.2	36.8 (0.209)
Controls				
7	No inflammatory disease	36.7	37.5	37.0 (0.228)
8	No inflammatory disease	36.4	37.2	36.6 (0.148)
9	RA^d^ in remission	36.3	36.9	36.5 (0.127)
10	No inflammatory disease	36.4	36.8	36.6 (0.094)
11	No inflammatory disease	36.8	37.3	37.0 (0.094)
12	No inflammatory disease	36.6	37.1	36.8 (0.133)
13	SpA in remission	36.3	36.8	36.6 (0.163)
	Control group average	36.5	37.1	36.7 (0.141)

a For each participant, minimum (nadir), maximum (peak), and mean (SD) of CBT were calculated based on the temperature measurements collected between 10 PM and 7 AM the following day.

b VEXAS: vacuoles, E1 enzyme, X-linked, autoinflammatory, somatic.

c SpA*:* spondyloarthritis.

d RA*:* rheumatoid arthritis.

### Predictive Accuracy of Multimodal, Noninvasive Inflammatory Markers

To assess the predictive accuracy of the noninvasive methods, we identified the optimal subset of multimodal, noninvasive biomarkers for predicting serum CRP levels using the all-subset regression method (Table 4). We considered urine CRP, creatinine-normalized urine CRP, and saliva CRP measurements due to their robust correlations with serum CRP. To ensure analytical validity and consistency, models were developed using data from 15 participants with complete urine and saliva measurements, excluding 4 urine samples below the assay’s lower limit of quantification. Among the possible covariates, the best subset of the model achieved an adjusted *R*^2^ value of 0.761, indicating that 76.1% of the variance in the serum CRP is collectively explained by salivary CRP and absolute urine CRP, surpassing the performance of single modality approaches. Serum CRP and creatinine-normalized urine CRP yielded an adjusted *R*^2^ value of 0.747. When urine CRP, creatinine-normalized urine CRP, and saliva CRP were modeled separately, the *R*^2^ value ranged from 0.52 to 0.61.

**Table 4.** All-subset regression models evaluating combinations of multimodal, noninvasive inflammatory biomarkers for predicting serum C-reactive protein (CRP) levels^a^.

Regression model		Adjusted *R*^2^
Model 1	Serum CRP~ sa-CRP^b^	0.518
Model 2	Serum CRP~u-CRP^c^	0.608
Model 3	Serum CRP~u-CRP/u-Cr^d^	0.513
Model 4^e^	Serum CRP~saCRP+u-CRP	0.761
Model 5	Serum CRP~saCRP+u-CRP/u-Cr	0.747
Model 6	Serum CRP~uCRP+u-CRP/u-Cr	0.691
Model 7	Serum CRP~saCRP+uCRP+u-CRP/u-Cr	0.745

a Each model includes noninvasive markers as predictors: sa-CRP, u-CRP, and u-CRP/u-Cr.

b sa-CRP: saliva C-reactive protein.

c u-CRP: urine C-reactive protein.

d u-CRP/uCr: creatinine-normalized urine C-reactive protein.

e Model 4, which includes both sa-CRP and u-CRP, demonstrated the highest explanatory power.

Participant-level analysis revealed that this performance enhancement can be attributed to the complementary roles of saliva and urine, particularly in cases where 2 noninvasive methods display discrepancies in predicting accuracy (Table S3 in Multimedia Appendix 1). In the case of a patient with giant cell arteritis, for example, saliva CRP was more accurate than urine CRP in predicting serum CRP levels. In contrast, a patient with seropositive erosive RA showed that urine CRP levels predicted serum CRP levels more accurately than saliva. The multimodal approach was also effective in handling outliers, such as a control patient with adult-onset Still disease in remission, who exhibited markedly high saliva CRP values despite low serum and urine CRP levels. Integration of urine and saliva CRP measurements substantially mitigated discrepancies and significantly improved prediction accuracy, suggesting a potential for practical and noninvasive solutions for CRP detection.

### Patient Preference

We obtained participants’ preferences for various biospecimen sampling methods, ranked from most to least preferred. Among all participants (N=19), the sweat patch and urine test were rated as the most acceptable methods, with mean scores of 2.37 (SD 1.30) and 2.42 (SD 1.54), respectively. Preferences were consistent across groups. For the sweat patch, the mean scores were identical in the SI and control groups at 2.3 (SD 1.34 and 1.33, respectively); for the urine test, mean ratings were slightly higher in the SI group at 3.0 (SD 1.76) compared to 1.78 (SD 0.97) in controls.

Moderately preferred methods included FeNO test, saliva test, and CBT sensor, which yielded mean scores of 3.63 (SD 1.86), 3.68 (SD 1.25), and 4.08 (SD 1.38), respectively, across all participants. The FeNO test was rated similarly by both groups, with means of 3.9 (SD 1.52) in the SI group and 3.3 (SD 2.24) in controls. Saliva sampling received mean scores of 3.3 (SD 1.16) in the SI group and 4.11 (SD 1.27) among controls. CBT sensor was rated with means of 3.86 (SD 1.57) in the SI group and 4.33 (SD 1.21) in controls.

The least preferred methods were blood and stool tests. The blood test received a mean score of 4.47 (SD 2.04) overall, with the SI group rating it at 4.2 (SD 2.35) and the control group slightly higher at 4.78 (SD 1.72). The stool test was rated lowest across all sampling methods, with a mean of 5.84 (SD 1.46).

## Discussion

### Principal Findings

This is the first study to assess the accuracy of a multimodal, noninvasive assessment of inflammatory biomarkers compared to the gold-standard serum CRP in a cohort with and without SI. Our main findings revealed that CRP levels in urine and saliva were significantly elevated in patients with SI and exhibited strong correlations with serum CRP regardless of the underlying diagnosis. Notably, the combination of urine and saliva CRP achieved high predictive accuracy for serum CRP with improvements of 15%‐24% over single-modality models. The multimodal approach effectively addressed outliers and discrepancies occurring in the single modalities. The measurements in urine and saliva demonstrated high detection rates and were strongly favored over blood tests by participants. These results highlight urine and saliva as promising alternative biofluids for CRP assessments, with significant implications for digitization.

Establishing these relationships would provide the basis for digitizing measurement for serum biomarkers, such as for CRP, contributing to the creation of a digital surrogate biomarker. Once digitized, this approach could lead to the development of a scalable, cost-efficient sensor, allowing a timely and rapid monitoring of inflammatory activity. This advancement is particularly relevant, given the ever-rising incidence of systemic inflammatory conditions and the urgent need for precise and remote monitoring tools.

### Comparison With Prior Research

Previous literature regarding noninvasive detection and digitization of inflammatory biomarkers is scarce. Few studies showed that CRP might be detected in urine; however, they were mostly concerning urologic conditions and did not use the urine/creatinine ratio to overcome urine dilution variability [10,39]. An additional study suggested that CRP excretion in urine is dependent on the levels of proteinuria [40]. In our study, we found that patients in the SI and control groups had similar levels of proteinuria and albuminuria.

The first evidence regarding the detection of CRP in saliva, reported in veterinary medicine, demonstrated a correlation between serum and salivary CRP in dogs comparable to our findings. In humans, a wide range of correlation coefficients between serum and salivary CRP levels across various conditions was reported [17]. This variability may stem from several challenges, with the most relevant being the lack of standardized collection techniques, the impact of local microtrauma, variations in salivary flow rates, and dilution levels. We aimed to mitigate these issues in our study by implementing the same technique of collection and requirements regarding eating and drinking as well as collecting information about known gingival inflammatory diseases.

### Strengths and Limitations

A key strength of this study lies in the innovative approach of a multimodal testing of noninvasive measurements in the context of SI irrespective of diagnosis. Moreover, the methods proposed are straightforward and easily adaptable for further testing and validation, ensuring their potential for broader application, integration into future research, clinical practice, and digitization for remote monitoring. Additionally, it prioritizes patient-centered care by incorporating patient preferences, making the findings highly relevant to clinical practice.

However, the generalizability of our findings is limited by the small size of the cohort and the lack of longitudinal data. Another limitation refers to the detection of inflammatory markers in sweat. Finally, our recruitment and study procedure were based on a single university hospital located in Zurich, Switzerland. These limitations do not compromise the interpretability of the main results regarding a high detection rate and good correlation of urine and saliva measurements with serum CRP.

### Suggestions for Future Sensor Development

To achieve the digitization of CRP and efficient sensor development, we highlight several key considerations based on the findings of our study. First, sensor accuracy and reliability must be ensured, considering the extremely low concentration of CRP and its detection rate in biofluids at the picomolar level. While biofluids such as urine, saliva, and sweat contain a variety of biochemical molecules and clinically relevant biomarkers, the concentration of these markers is generally much lower in noninvasive biofluids than in blood. Due to the nature of the feasibility study, we used immunoassay analyses tailored to each biofluid, following methodologies established in previous studies. For the sensor to be scalable, developing cost-efficient yet sensitive analytic methods can be a major challenge.

Second, we need to consider interindividual variability and diurnal fluctuations of noninvasive biofluids [17,41]. The excretion of these biofluids is subject to influences from interpersonal or environmental factors. In our study, we used standardized protocols, requiring participants to abstain from food and substances prior to sample collection to minimize these variables. We also collected comprehensive demographic and health data through questionnaires. For future sensor developments, the collection of this contextual information is crucial, and its digitization could improve efficiency in real-world applications.

Third, patient preference and acceptability play a pivotal role in the practical implementation. In our qualitative results, participants ranked urine, saliva, and sweat as the top 3 most preferred methods due to their accessibility and the ability to perform stress-free, repeated sampling. This finding suggests that these methods could be successfully implemented with high acceptability in real-world settings, as they reduce the time and pain associated with traditional blood-based testing. Furthermore, participants did not find multimodal biofluid collection burdensome but easy to perform with minimal preparation required for testing, which aligns with a patient-centered approach that promotes autonomy.

Finally, the different biofluids may require different implementation strategies. In our study, the detection of inflammatory markers in sweat (passive sweat collected using sweat patches) was particularly challenging. Several factors may have contributed to this, including a short exposure window (48 hours as opposed to 72 hours in previous studies) and the relatively older age of study participants may have led to insufficient passive sweat excretion [42]. Additionally, the prolonged storage of sweat patches due to batch analysis design could have resulted in protein degradation. A recent study has demonstrated the real-time, noninvasive CRP monitoring using a wireless sweat sensor in both healthy individuals and patients with heart failure in a laboratory setting [20]. While our study did not yield similar results, the high preference for sweat collection among participants, easier accessibility, and reduced risk of contamination suggest that it still holds potential for the real-time assessment [43].

### Conclusions

Our feasibility study demonstrates the potential of urine and saliva CRP as surrogate markers for SI and holds significant digitization potential. Beyond their immediate clinical relevance, this discovery underscores the potential of these biofluids as valuable targets for developing novel sensor technologies, which paves the way toward scalable health monitoring solutions and personalized patient-centered care. Digital biomarkers could enhance health equity and accessibility, especially in rural areas or underserved communities, thus reducing treatment gaps and fostering inclusive patient care. Future studies focusing on longitudinal follow-up and populations with systemic inflammatory disorders and comorbidities will be crucial for validating and generalizing our findings.

## Supplementary material

10.2196/77108 Multimedia Appendix 1 Participant eligibility, study procedure, survey questionnaire, and additional analyses.

## Abbreviations

CBT: core body temperature
CRP: C-reactive protein
FeNO: fractional exhaled nitric oxide
IL: interleukin
RA: rheumatoid arthritis
SI: systemic inflammation
STROBE: Strengthening the Reporting of Observational Studies in Epidemiology
TNF: tumor necrosis factor
USZ: University Hospital Zurich
