# Supplementary material for: Multimodal Noninvasive Assessment of C-Reactive Protein for Systemic Inflammation in Adults: Cross-Sectional Study
Source: JMIR Form Res. 2025 Aug 26;9:e77108. doi: 10.2196/77108 (PMC12379750; doi:10.2196/77108)
Supplement: Multimedia Appendix 1 [file formative-v9-e77108-s001.docx]

**Figure S1.** Participant flow diagram.


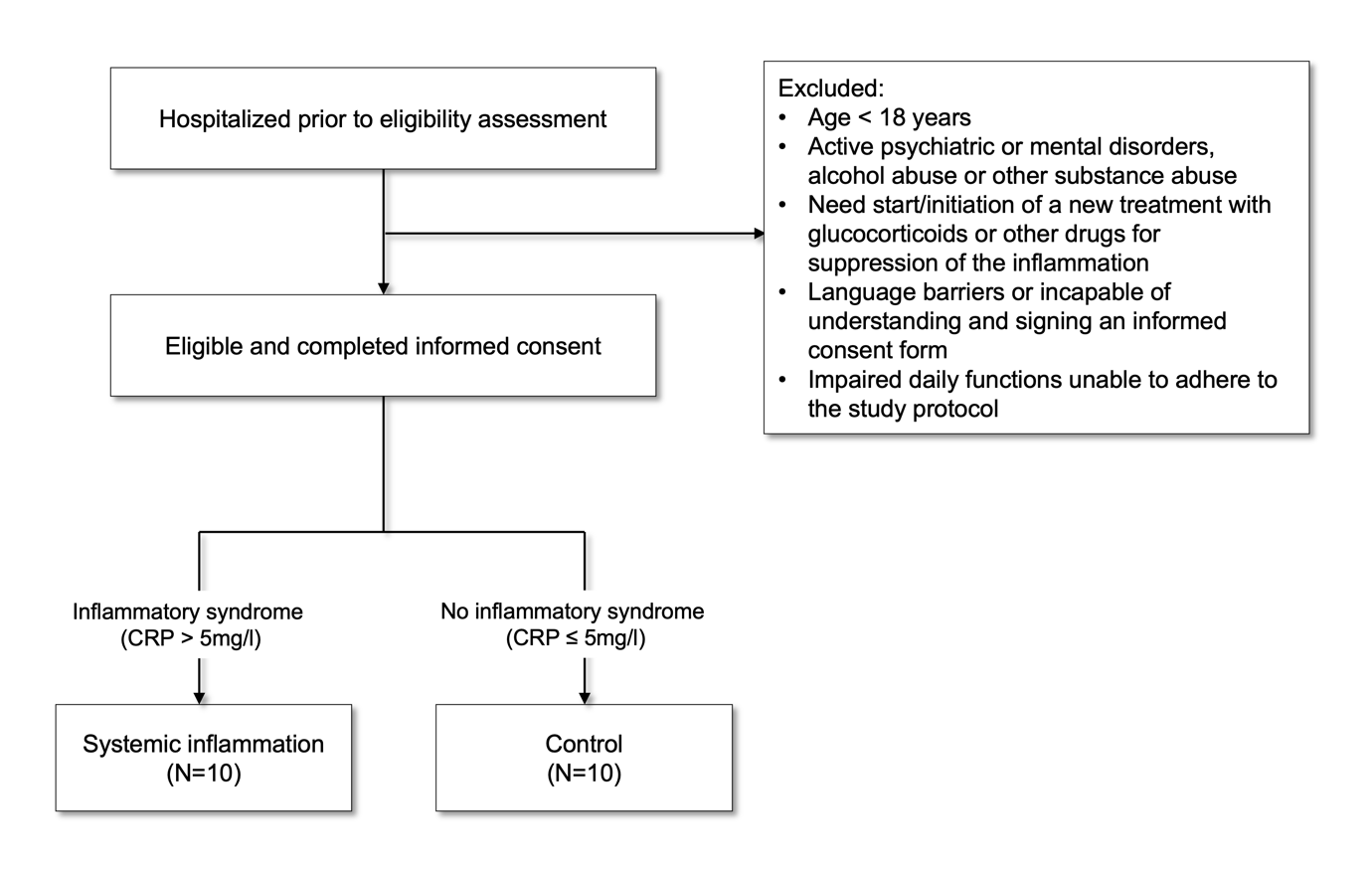


**Figure S2.** Overview of the study procedure.


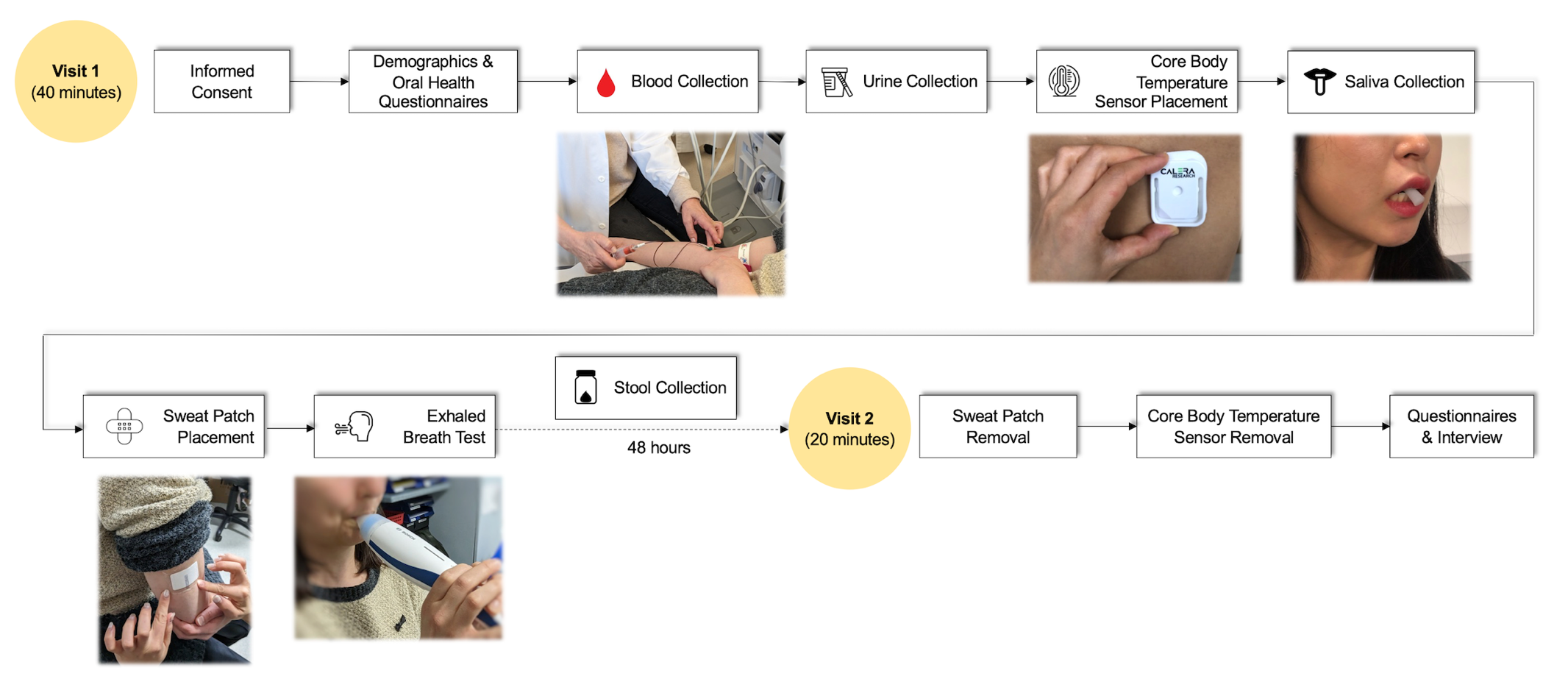


**Figure S3.** Distributions of inflammatory cytokine levels in sweat and urine among participants with systemic inflammation (SI) and controls. Biomarkers with a detectability rate above 70% are shown. Boxes indicate the interquartile range (IQR), and horizontal line within each box represents the median, and whiskers denote 1.5xIQR. Dots represent outliers.


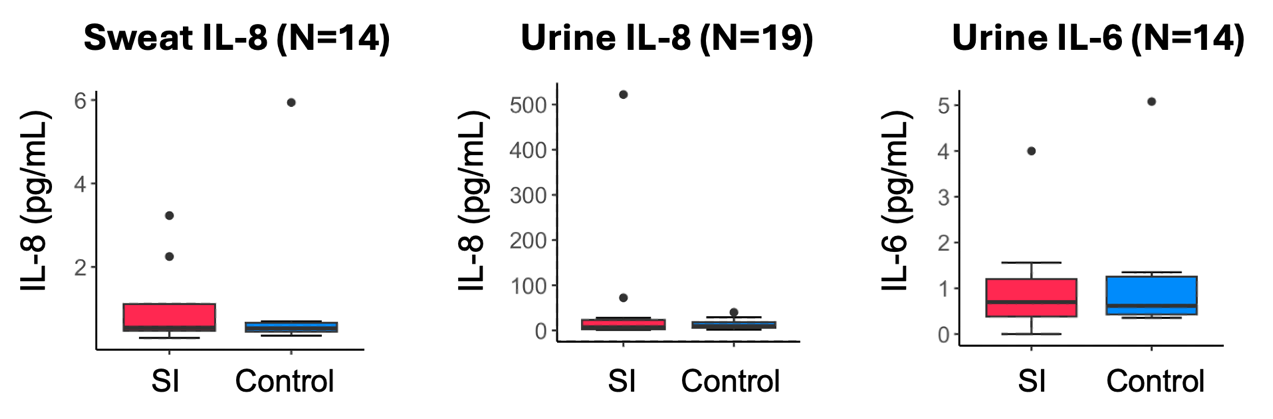


**Figure S4.** Core body temperature trajectories from patients with SI and controls. **a-f** SI patients. Individual diagnosis includes **a** Adult onset Still disease (CRP=216.0 mg/L). **b** VEXAS Syndrome (CRP=81.0 mg/L). **c** Peripheral spondyloarthritis (CRP=32.0 mg/L). **d** Undifferentiated seronegative polyarthritis (CRP=20.0 mg/L). **e** Giant cell arteritis (CRP=16.0 mg/L). **f** Unclear diagnostic delay multiple sclerosis (CRP=5.6 mg/L). **g-m** Controls. Hourly mean value is depicted. Grey region indicates night hours (10pm-7am).


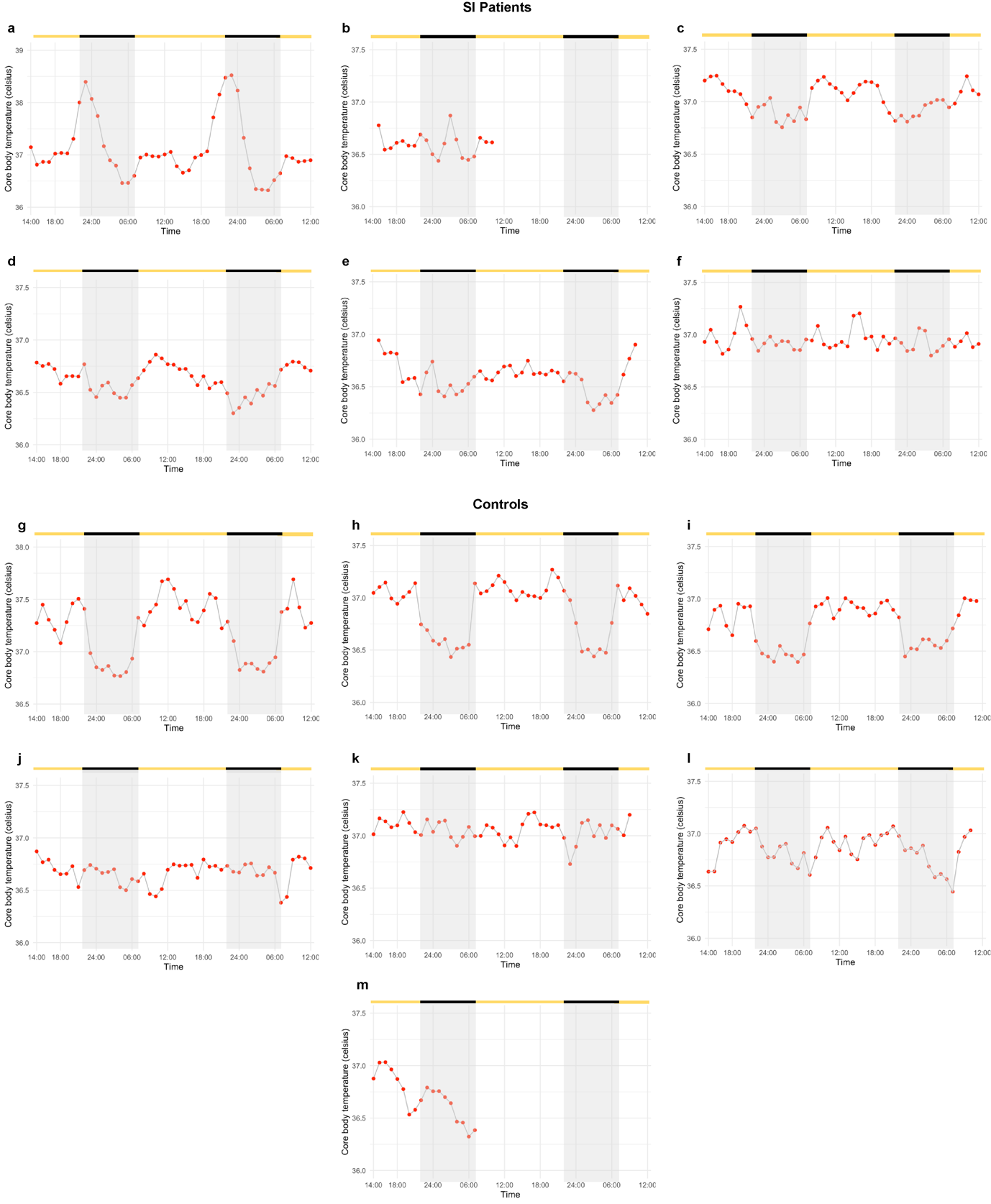


**Table S1.** Comparison of urinary albumin and total protein levels between participants with systemic inflammation (SI) and controls. Values are presented as median (interquartile range, IQR). Between-group comparisons were performed using the Mann–Whitney U test.

| **Median (IQR) or N (%)** | **SI (N=10)** | **Control (N=9)** | **p-value** |
| --- | --- | --- | --- |
| Urine albumin (mg/mmol) | 3.0 (3.0, 4.3) | 8.2 (3.0, 14.6) | 0.101 |
| Urine total protein (g/mmol) | 0.04 (0.04, 0.06) | 0.06 (0.04, 0.08) | 0.127 |

**Table S2.** Detection rates and group comparisons of multi-modal inflammatory biomarkers across serum, urine, saliva, sweat, exhaled breath, and stool samples. Detection rates are reported as number of detected samples over total tested. Biomarker concentrations are presented as median (interquartile range, IQR). Between-group comparisons of participants with systemic inflammation (SI) and controls were performed using the Mann–Whitney U test. CRP, C-reactive protein; IL, interleukin; TNF-α, tumor necrosis factor-alpha; FeNO, fractional exhaled nitric oxide.

| **Sample** | **Biomarker (Unit)** | **Detection Rate (Detected/Total)** | **SI  Median (IQR)** | **Control Median (IQR)** | **p-value** |
| --- | --- | --- | --- | --- | --- |
| Serum | CRP (mg/L) | 100% (19/19) | 18  (9.3, 68.8) | 1.3  (1.2, 1.8) | **0.0002** |
| Urine | CRP  (pg/mL) | 79% (15/19) | 23318  (14486, 40774) | 6503  (3121, 8927) | **0.0002** |
| Urine-Cr normalized | CRP  (μg/mmol) | 79% (15/19) | 4.5  (4.15, 10.3) | 0.69  (0.24, 1.39) | **0.001** |
| Saliva | CRP (pg/mL) | 100% (19/19) | 4910  (2735, 13275) | 473  (309, 700) | **0.001** |
| Sweat | CRP | 0% (0/19) | - | - | - |
| Serum | IL-6 (pg/mL) | 100% (19/19) | 2.58  (1, 10.1) | 1  (1, 1) | **0.015** |
|  | IL-8 (pg/mL) | 100% (19/19) | 23.3  (16.2, 38.0) | 27.7  (20.8, 31.0) | 0.84 |
|  | IL-10 (pg/mL) | 100% (19/19) | 1.0  (0.84, 1.64) | 0.97  (0.77, 1.6) | 0.51 |
|  | IL-1β (pg/mL) | 100% (19/19) | 1  (1,1) | 1 (1,1) | 0.34 |
|  | TNF-α (pg/mL) | 100% (19/19) | 8.85  (6.72, 9.44) | 5.83  (3.4, 7.17) | **0.017** |
| Urine | IL-6 (pg/mL) | 74% (14/19) | 0.70  (0.38, 1.20) | 0.62  (0.43, 1.25) | 0.71 |
|  | IL-8 (pg/mL) | 100% (19/19) | 7  (2.53, 23.2) | 9.57  (5.81, 18.3) | 0.6 |
|  | IL-10 (pg/mL) | 5% (1/19) | 0.21  (0.21, 0.21) | - | - |
|  | IL-1β (pg/mL) | 21% (4/19) | 1.33  (0.88, 8.72) | 0.57 (0.57, 0.57) | 1.0 |
|  | TNF-α (pg/mL) | 5% (1/19) | 0.25 (0.25, 0.25) | - | - |
| Sweat | IL-6 (pg/mL) | 42% (8/19) | 0.41  (0.41, 0.42) | 0.37  (0.36, 0.40) | 0.43 |
|  | IL-8 (pg/mL) | 74% (14/19) | 0.54  (0.47, 1.11) | 0.53  (0.45, 0.66) | 0.95 |
|  | IL-10 (pg/mL) | 11% (2/19) | 0.20  (0.20, 0.20) | 0.2 (0.2, 0.2) | - |
|  | IL-1β (pg/mL) | 32% (6/19) | 0.46  (0.44, 0.77) | 0.79  (0.57 1.81) | 1.0 |
|  | TNF-α  (pg/mL) | 47% (9/19) | 0.30  (0.29, 0.31) | 0.35 (0.31, 0.37) | 0.2 |
| Exhaled Breath | FeNO (ppb) | 42% (8/19) | 21  (20, 28) | 11  (9,12) | **0.036** |
| Stool | Calprotectin (ug/g) | 95% (18/19) | 62.5  (17.5, 97.2) | 103  (88, 129) | 0.12 |

Bold text indicates p-value < 0.05.

**Table S3.** Ranking of CRP concentrations across serum, urine, and saliva samples in individual participants with systemic inflammation (SI) and controls. Participants are ordered by descending serum CRP values. For each sample type, the measured CRP concentration and corresponding within-cohort rank (from highest to lowest) are shown. Asterisks (*) indicates a rank difference greater than 3 between serum and the respective non-invasive measure. CRP, C-reactive protein; SpA, spondyloarthritis; RA, rheumatoid arthritis; PVNS, pigmented villonodular synovitis.

|  | Diagnosis | Serum CRP (mg/L) | Serum Rank | Urine CRP (pg/mL) | Urine Rank | Saliva CRP (pg/mL) | Saliva Rank |
| --- | --- | --- | --- | --- | --- | --- | --- |
| SI Patients | Adult onset Still disease | 216 | 1 | 65829 | 2 | 27700 | 1 |
|  | Transient polyarthritis | 155 | 2 | 158607 | 1 | 13300 | 3 |
|  | VEXAS Syndrome | 81 | 3 | 40774 | 3 | 5830 | 5 |
|  | Peripheral SpA | 32 | 4 | 31397 | 4 | 3470 | 7 |
|  | Undifferentiated seronegative polyarthritis | 20 | 5 | 23318 | 5 | 3990 | 6 |
|  | Giant cell arteritis | 16 | 6 | 7273 | 12* | 2490 | 8 |
|  | PVNS | 7.4 | 7 | 13590 | 8 | 13200 | 4 |
|  | Seropositive erosive RA | 7.1 | 8 | 14486 | 7 | 259 | 14* |
|  | Inflammatory syndrome likely linked to multiple sclerosis | 5.6 | 9 | 16392 | 6 | 1330 | 9 |
| Controls | No inflammatory disease | 2.5 | 10 | 9144 | 10 | 1330 | 10 |
|  | Adult onset Still disease in remission | 1.8 | 11 | 10159 | 9 | 17600 | 2* |
|  | No inflammatory disease | 1.3 | 12 | 447 | 15 | 125 | 15 |
|  | No inflammatory disease | 1.2 | 13 | 2585 | 14 | 309 | 13 |
|  | SpA in remission | 0.9 | 14 | 4729 | 13 | 700 | 11 |
|  | No inflammatory disease | 0.7 | 15 | 8277 | 11* | 473 | 12 |

**Table S4.** Individual-level CRP levels in serum and saliva from 4 patients with non-detectable urine CRP values

|  | Diagnosis | Serum CRP (mg/L) | Saliva CRP (pg/mL) |
| --- | --- | --- | --- |
| SI Patient | Mixed crystal-induced arthropathy CPPD and gout | 15.0 | 25700 |
| Controls | No inflammatory disease | 1.4 | 597 |
|  | No inflammatory disease | 2.3 | 364 |
|  | RA in remission | 1.3 | 126 |

**Table S5.** Inflammatory diagnoses in the control group to assess non-invasive methods for detection of inflammatory parameters

| Main Diagnosis | Count | Serum CRP (mg/L) | Medication |
| --- | --- | --- | --- |
| Adult Still syndrome | 1 | 0.6 | Canakinumab,  Methotrexate |
| Spondyloarthritis (axial and peripheral) | 1 | 1.9 | Golimumab |
| Rheumatoid arthritis | 1 | 0.6 | Treatment stopped,  only coxibes |

**Methods S1.** Baseline Questionnaire

| **Demographics** | |
| --- | --- |
| 1. How old are you? | _________ years old |
| 1. What is your sex?   ☐ Male | ☐ Female |
| 1. What is your race? ☐ African American   ☐ Asian | ☐ Caucasian  ☐ Other: _________________________ |
| 1. What is your marital status? ☐ Single (never married)   ☐ Married  ☐ Divorced | ☐ Separated  ☐ Widowed  ☐ Other: _________________________ |
| 1. What is your work status? ☐ Full-time   ☐ Retired  ☐ Not employed | ☐ Part-time  ☐ Self-employed  ☐ Other: _________________________ |
| 1. What is the highest degree or level of education you have completed? | |
| ☐ Incomplete high(secondary) school  ☐ Completed high(secondary) school  ☐ Bachelor’s degree | ☐ Master’s degree  ☐ Ph.D. or higher  ☐ Other: _________________________ |
| **Oral Health** | |
| 1. Are you fasted? (No eating or drinking except water in the last 2 hours) | |
| ☐ Yes  If no, list items that you ate or drank:  _____________________________________ | ☐ No |
| 1. Have you brushed your teeth or used mouthwash in the last 2 hours?   ☐ Yes ☐ No | |
| 1. Have you chewed gum in the last 2 hours?   ☐ Yes ☐ No | |
| 1. Have you smoked in the last 2 hours?   ☐ Yes ☐ No | |
| 1. Have you drunken alcohol in the last 24 hours?   ☐ Yes ☐ No | |
| 1. Have you ever noticed bleeding from your gums when not brushing in the past 24 hours?   ☐ Yes ☐ No | |
| 1. Do you currently have any of these oral diseases? periodontal disease and gingivitis, periodontitis, autoimmune, infectious, musculoskeletal, or malignant disease, and recent operation or trauma   ☐ Yes ☐ No  If yes, are you currently receiving treatment for it?  ☐ Yes ☐ No | |
| **Previous Hospitalizations and Surgeries** | |
| 1. Have you been hospitalized in the last 1 year due to an inflammatory disease?   ☐ Yes ☐ No | |
| 1. If “Yes”, what is the total number of hospital stays in the last 1 year?    ______________________ Days | |
| 1. Have you had any surgeries in the last 1 year?   ☐ Yes ☐ No | |
| 1. If "Yes", how many the total number of surgeries did you receive in the last 1 year?    ______________________ | |

**Methods S2.** Questionnaire on Assessment of Sampling Methods

*Instruction to patients: Please indicate your agreement with the attributes by ticking the circle that most closely reflects your impression of the measurement method you just completed.*

| **Blood** | **Q1** | Enjoyable | ◯ ◯ ◯ ◯ ◯ | Annoying |
| --- | --- | --- | --- | --- |
|  | **Q2** | Simple | ◯ ◯ ◯ ◯ ◯ | Complicated |
|  | **Q3** | Efficient | ◯ ◯ ◯ ◯ ◯ | Inefficient |
|  | **Q4** | Less burdensome | ◯ ◯ ◯ ◯ ◯ | Burdensome |
|  | **Q5** | Easy to learn | ◯ ◯ ◯ ◯ ◯ | Difficult to learn |
| **Urine** | **Q1** | Enjoyable | ◯ ◯ ◯ ◯ ◯ | Annoying |
|  | **Q2** | Simple | ◯ ◯ ◯ ◯ ◯ | Complicated |
|  | **Q3** | Efficient | ◯ ◯ ◯ ◯ ◯ | Inefficient |
|  | **Q4** | Less burdensome | ◯ ◯ ◯ ◯ ◯ | Burdensome |
|  | **Q5** | Easy to learn | ◯ ◯ ◯ ◯ ◯ | Difficult to learn |
| **Sweat patch** | **Q1** | Enjoyable | ◯ ◯ ◯ ◯ ◯ | Annoying |
|  | **Q2** | Simple | ◯ ◯ ◯ ◯ ◯ | Complicated |
|  | **Q3** | Efficient | ◯ ◯ ◯ ◯ ◯ | Inefficient |
|  | **Q4** | Less burdensome | ◯ ◯ ◯ ◯ ◯ | Burdensome |
|  | **Q5** | Easy to learn | ◯ ◯ ◯ ◯ ◯ | Difficult to learn |
| **Saliva** | **Q1** | Enjoyable | ◯ ◯ ◯ ◯ ◯ | Annoying |
|  | **Q2** | Simple | ◯ ◯ ◯ ◯ ◯ | Complicated |
|  | **Q3** | Efficient | ◯ ◯ ◯ ◯ ◯ | Inefficient |
|  | **Q4** | Less burdensome | ◯ ◯ ◯ ◯ ◯ | Burdensome |
|  | **Q5** | Easy to learn | ◯ ◯ ◯ ◯ ◯ | Difficult to learn |
| **Exhaled  breath** | **Q1** | Enjoyable | ◯ ◯ ◯ ◯ ◯ | Annoying |
|  | **Q2** | Simple | ◯ ◯ ◯ ◯ ◯ | Complicated |
|  | **Q3** | Efficient | ◯ ◯ ◯ ◯ ◯ | Inefficient |
|  | **Q4** | Less burdensome | ◯ ◯ ◯ ◯ ◯ | Burdensome |
|  | **Q5** | Easy to learn | ◯ ◯ ◯ ◯ ◯ | Difficult to learn |
| **Stool** | **Q1** | Enjoyable | ◯ ◯ ◯ ◯ ◯ | Annoying |
|  | **Q2** | Simple | ◯ ◯ ◯ ◯ ◯ | Complicated |
|  | **Q3** | Efficient | ◯ ◯ ◯ ◯ ◯ | Inefficient |
|  | **Q4** | Less burdensome | ◯ ◯ ◯ ◯ ◯ | Burdensome |
|  | **Q5** | Easy to learn | ◯ ◯ ◯ ◯ ◯ | Difficult to learn |
| **Core body temperature sensor** | **Q1** | Enjoyable | ◯ ◯ ◯ ◯ ◯ | Annoying |
|  | **Q2** | Simple | ◯ ◯ ◯ ◯ ◯ | Complicated |
|  | **Q3** | Efficient | ◯ ◯ ◯ ◯ ◯ | Inefficient |
|  | **Q4** | Less burdensome | ◯ ◯ ◯ ◯ ◯ | Burdensome |
|  | **Q5** | Easy to learn | ◯ ◯ ◯ ◯ ◯ | Difficult to learn |

**Methods S3.** Questionnaire on Preference for Sampling Methods

*Q1. Based on your experience in this study, please rank each of the following measurement methods in order of preference (1 - most preferred, 7 - least preferred). Please leave the box blank if you did not participate in the measurements.*

**Blood**

**Urine**

**Sweat
patch**

**Saliva**

**Exhaled
Breath**

**Stool**

**Core body
temperature
sensor**

*
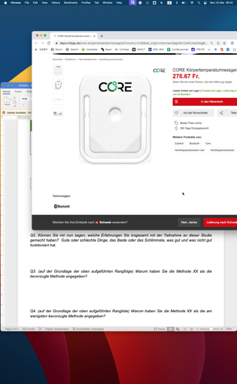
*

*
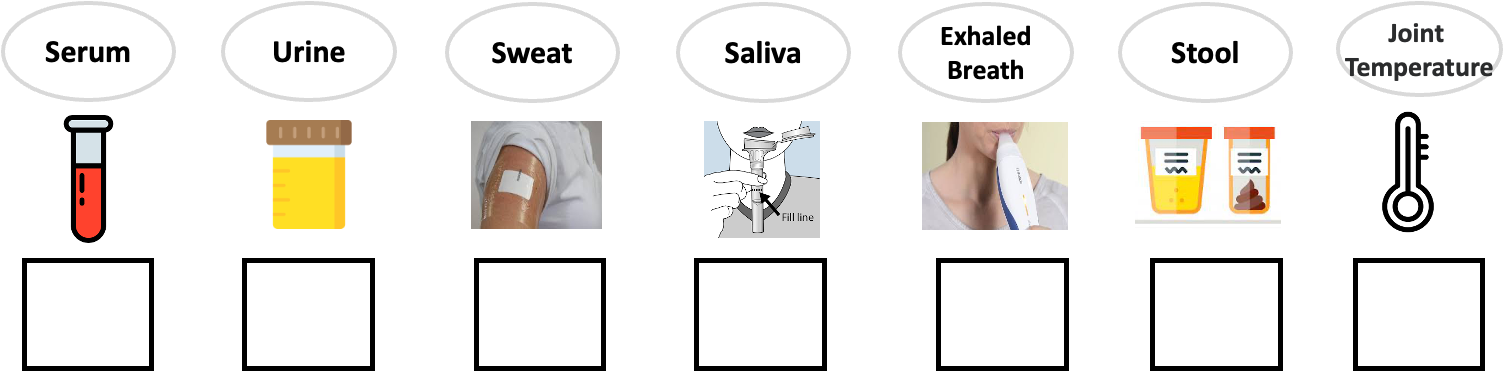
*

*Q2. Why did you choose this order? Please explain.*

*Q3. Could you tell me about your overall experience in participating in this study? Good or bad things, best or worst, what worked well and what did not work well.*

*Q4. Why did you decide to participate in this study?*

*Q5. Any other comments to share?*
